# Activation of Insula‐Accumbal Projection Neurons Is Required for Relapse‐Like Behaviour Following Opioid Self‐Administration

**DOI:** 10.1111/adb.70118

**Published:** 2026-01-12

**Authors:** Rachel E. Clarke, Bayleigh E. Pagoota, Isabella E. Dinu, Jacqueline E. Paniccia, Anna C. Tsyrulnikov, Annaka M. Westphal, Jade Baek, Michael D. Scofield, James M. Otis

**Affiliations:** ^1^ Department of Neuroscience Medical University of South Carolina Charleston South Carolina USA; ^2^ Anesthesiology and Perioperative Medicine Medical University of South Carolina Charleston South Carolina USA; ^3^ Hollings Cancer Center Medical University of South Carolina Charleston South Carolina USA; ^4^ Ralph H. Johnson VA Medical Center Charleston South Carolina USA

**Keywords:** insular cortex, nucleus accumbens, opioid, optogenetics, relapse, self‐administration

## Abstract

The insular cortex (IC) is known to underlie drug seeking and relapse for multiple drug classes, yet the precise role the IC plays in opioid use disorder (OUD) remains unclear. In preclinical models of OUD, inhibition of the IC has produced conflicting results, such that in some cases the IC seems to promote opioid seeking whereas in others the IC seems to blunt opioid seeking. These results may be related to the heterogeneity of cortical output circuits, which can have opposing functions despite their relative proximity. Thus, here we examined the role of a specific IC output circuit, from the anterior IC (aIC) to the nucleus accumbens core (NAcc), for opioid seeking. We find in mice that following 14 days of heroin self‐administration and 3 days of forced abstinence, optogenetic inhibition of aIC➔NAcc terminals suppresses context‐associated opioid seeking. Furthermore, the same manipulation attenuates cued opioid seeking following extinction training. Importantly, we observed no effect of aIC➔NAcc terminal inhibition on sucrose seeking. Together, our results reveal that the IC selectively controls opioid seeking through a discrete population of NAcc projecting neurons, providing the first evidence for a projection‐specific role of IC circuitry in opioid seeking and relapse.

## Abstract 2

The aIC has been implicated in opioid‐related behaviours, but the function of output‐defined aIC projection neurons for opioid seeking remains unknown. Using optogenetics we find that inhibition of activity in aIC➔NAcc projection neurons prevents context‐related and cue‐induced heroin seeking, whereas sucrose seeking is unaffected. Our results reveal that the aIC controls opioid seeking through a discrete population of NAcc projecting neurons, providing the first evidence for a role of the aIC➔NAcc projection in opioid seeking and relapse.

## Graphical abstract

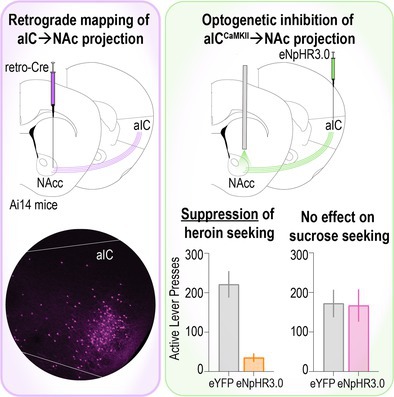

## 1 Introduction

The insular cortex (IC) serves as a critical hub where external sensory information converges with internal physiological and emotional states [1]. In the clinical setting, human neuroimaging studies demonstrate damage to the IC results in rapid and sustained disruption of nicotine craving and use [2]. While these data support a role for the IC in nicotine addiction, whether the IC similarly regulates opioid craving and relapse susceptibility remains unknown.

The IC is anatomically complex, with input and output connectivity varying dramatically along the rostro‐caudal axis of the IC [3, 4], dividing the IC into distinct subregions. The posterior IC (pIC) receives significant inputs from sensory, limbic and visceral regions [3, 4] and is thought to directly sense physiological state, relaying this information to the anterior IC (aIC) [1, 5]. The aIC then integrates these interoceptive signals with higher‐order cognitive and environmental information to appropriately guide motivated behaviour based on internal state [1, 5], likely via reciprocal connections with the thalamus, cortex and amygdala, as well as strong projections to the striatum [4]. Given the heterogeneous nature of IC circuitry, it is unsurprising than global silencing of the aIC has yielded mixed results. While some studies suggest the aIC exerts inhibitory control over opioid seeking [6, 7], others report that the aIC promotes seeking and relapse [8, 9, 10]. However, these nonspecific inhibition approaches do not consider the distinct functions of IC input and output circuits in guiding behaviour. In contrast, studies specifically targeting aIC outputs in the context of cocaine, amphetamine or alcohol seeking consistently demonstrate that aIC projections act to promote drug‐related behaviours [11, 12, 13, 14]. Given these findings in other drug classes, aIC output circuitry represents a compelling target for investigation in the context of opioid seeking.

The primary output of the aIC is the ventro‐lateral striatum, encompassing the nucleus accumbens core (NAcc), a key region involved in reward processing and motivated behaviour. Despite the significant contribution of the aIC➔NAcc projections to total aIC outputs [4], the role of this projection remains to be elucidated in the context of drug seeking. Only one study has tested the role of aIC➔NAcc neurons in opioid‐related behaviour and found this output to be necessary for expression of a morphine conditioned place preference (CPP) [15]. Notably, no studies have investigated whether aIC➔NAcc neurons promote volitional opioid seeking and regulate relapse in response to opioid‐paired cues.

Considering other corticostriatal circuits are critical regulators of opioid‐seeking behaviour [16, 17], here, we test the hypothesis that aIC➔NAcc neurons are (1) required for context‐associated opioid seeking following an acute involuntary abstinence period and (2) necessary for cued opioid seeking following extinction training. To ensure that any effects of aIC➔NAcc manipulation were specific to drug reward, we included a parallel sucrose self‐administration experiment to assess aIC➔NAcc function in natural reward seeking. Our results indicate that activation of aIC➔NAcc neurons selectively promotes drug, but not natural reward seeking, in response to contextual and reward‐paired cues. These data reveal a previously unknown role for aIC➔NAcc neuronal activity in driving opioid seeking and relapse.

## 2 Results

### 2.1 Lateral NAcc, Not Medial NAcc, Receives Dense aIC Input

**FIGURE 1:**
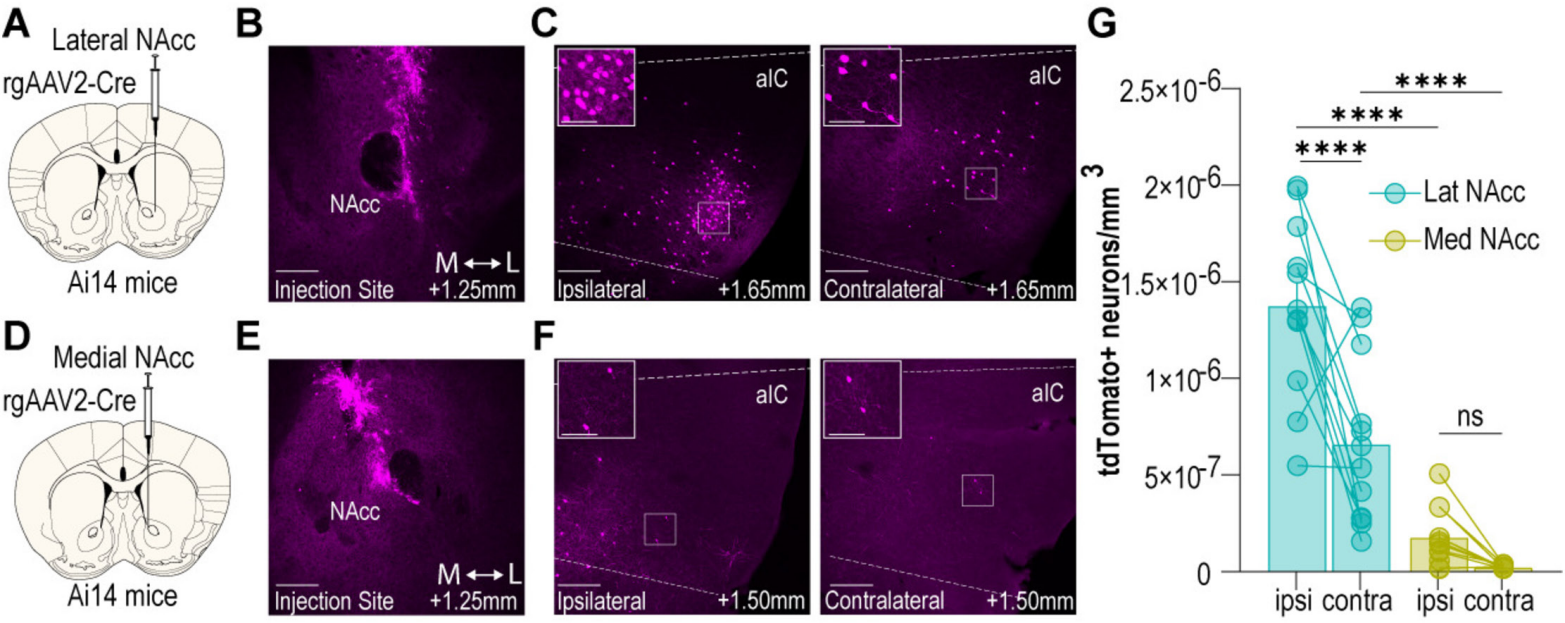
aIC sends dense projections to the lateral but not medial NAcc. (A) Schematic illustrating viral strategy and injection site for targeting lateral NAcc. (B) Representative image of lateral NAcc injection site with anterior–posterior position relative to bregma indicated in bottom right of figure. Scale bar = 300 μm. (C) Representative image of ipsilateral (left) and contralateral (right) aIC of animal with lateral NAcc injection with anterior–posterior position relative to bregma indicated in bottom right of figure. Scale bar = 300 μm. White box indicates area shown at higher magnification in insert. Inset scale bar = 100 μm. (D) Schematic illustrating viral strategy and injection site for targeting medial NAcc. (E) Representative image of medial NAcc injection site with anterior–posterior position relative to bregma indicated in bottom right of figure. Scale bar = 300 μm. (F) Representative image of ipsilateral (left) and contralateral (right) aIC of animal with medial NAcc injection site with anterior–posterior position relative to bregma indicated in bottom right of figure. Scale bar = 300 μm. White box indicates area shown at higher magnification in insert. Inset scale bar = 100 μm. (G) Quantification of tdTomato+ neurons per volume of tissue for ipsilateral and contralateral aIC from animals with lateral NAcc (cyan) or medial NAcc (yellow) (*n* = 12 sections from 2–3 animals per injection site; two‐way ANOVA, injection site effect: *F*_(1, 44)_ = 100.5, *p* < 0.0001, hemisphere effect: *F*_(1, 44)_ = 22.62, *p* < 0.0001, interaction *F*_(1, 44)_ = 9.399, *p* = 0.0037, Sidak's post hoc test, lateral injection site: ipsi versus contra: *p* < 0.0001, ipsilateral hemisphere: lat versus med: *p* < 0.0001, contralateral hemisphere: lat versus med *p* < 0.0001). Data are mean ± SEM ****p < 0.0001. aIC, anterior insular cortex; contra, contralateral: ipsi, ipsilateral; lat, lateral; med, medial; NAcc, nucleus accumbens core.

Previous studies report that the aIC projects primarily to the lateral region of the NAcc [4]; however, a direct comparison between aIC inputs to the medial and lateral NAcc has never been made. Therefore, to precisely characterize the projection patterns of the aIC to NAcc outputs, we assessed the number of aIC neurons that project to the medial versus the lateral region of the NAcc using viral‐assisted retrograde tracing in transgenic reporter mice (Figure 1). Using high‐resolution confocal microscopy and IMARIS image reconstruction, we found retrograde Cre injection to the lateral NAcc (Figures 1A,B and S1A,B) resulted in dense expression of tdTomato in the ipsilateral and contralateral hemispheres of the aIC (Figure 1C). In contrast, we observed sparse labelling of aIC cells when the medial NAcc was targeted (Figure 1D–F). Quantification of the number of tdTomato expressing neurons per volume of tissue in the aIC revealed that the aIC primarily projects to the lateral NAcc, with the ipsilateral projection displaying a greater number of tdTomato expressing cells versus the contralateral projection (Figure 1G). The results of this tracing study reveal that larger populations of aIC neurons project to the lateral NAcc compared to the medial NAcc. Considering this finding, we targeted aIC➔lateral NAcc projection neurons to determine whether this pathway functionally guides opioid‐seeking behaviour.

### 2.2 Optogenetic Inhibition of aIC ➔NAcc Terminals Supresses Heroin Seeking

**FIGURE 2:**
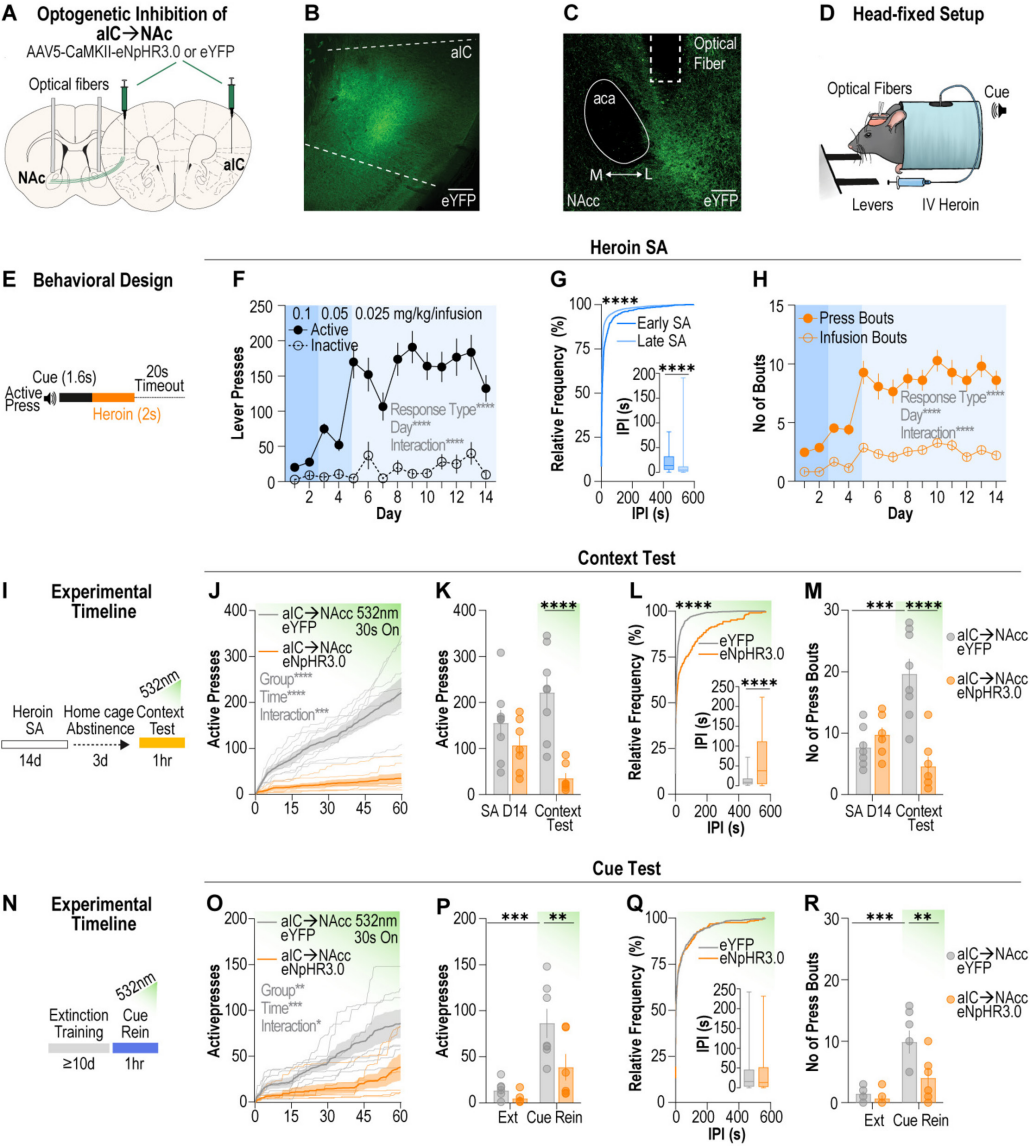
aIC➔NAcc neurons are necessary for reinstatement of heroin seeking. (A) Viral approach used to optogenetically inhibit aIC➔NAcc terminals. (B) Representative images showing eYFP expression in the aIC, scale bar = 300 μm and (C) eYFP expressing fibres in the NAcc with optical fibre placement, scale bar = 150 μm. (D) Schematic depicting head‐fixed behavioural apparatus. (E) Behavioural paradigm for intravenous head‐fixed heroin self‐administration. (F) Grouped data for acquisition of heroin self‐administration. Mice learn to discriminate between the active and inactive levers, with greater active lever presses across acquisition (*n* = 15 mice; mixed‐effects model, lever effect: *F*_(1, 28)_= 104.4, *p* < 0.0001, day effect: *F*_(6.423178.8)_ = 12.62, *p* < 0.0001, interaction: *F*_(6.423178.8)_ = 8.209, *p* < 0.0001). (G) Cumulative frequency distribution of interpress intervals from early (days 1–2) and late (days 13–14) heroin self‐administration (*n* = 15 mice; Kolmogorov–Smirnov test, Kolmogorov–Smirnov *D* = 0.3632, *p* < 0.0001). Inset shows box and whisker plots for interpress intervals for early and late sessions (*n* = 15 mice; Mann–Whitney test, Mann–Whitney *U* = 16 880, *p* < 0.0001). (H) Infusion bouts, defined as three or more infusions in a 2‐min period, and press bouts, defined as three or more lever presses with a 25‐s period, increased across heroin self‐administration (*n* = 15 mice; mixed‐effects model, lever effect: *F*_(1, 28)_= 144.4, *p* < 0.0001, day effect: *F*_(6.939193.2)_ = 17.27, *p* < 0.0001, interaction: *F*_(6.939193.2)_ = 5.317, *p* < 0.0001). (I) Experimental timeline for context test. (J–K) Inhibition of aIC➔NAcc terminals suppressed active lever pressing during the context test. (*n* = 7–8 mice per group; J: two‐way ANOVA, group effect: *F*_(1, 13)_=36.89, *p* < 0.0001, time effect: *F*_(1.418, 18.43)_ = 33.49, *p* < 0.0001, interaction: *F*_(1.418, 18.43)_ = 18.93, *p* = 0.0001; K: two‐way ANOVA, group effect: *F*_(1, 13)_=30.64, *p* = 0.0001, time effect: *F*_(1, 13)_= 0.0007614, *p* = 0.9318, interaction: *F*_(1, 13)_= 5.136, *p* = 0.0411, Sidak's post hoc test, context test: *p* = 0.0001). (L) Cumulative frequency distribution of interpress intervals from eYFP and eNpHR3.0 mice during the context test (*n* = 7–8 mice per group; Kolmogorov–Smirnov test, Kolmogorov–Smirnov *D* = 0.4789 *p* < 0.0001). Inset shows box and whiskers plot for interpress intervals during context test (*n* = 7–8 mice per group; Mann–Whitney test, Mann–Whitney *U* = 9635, *p* < 0.0001). (M) Inhibition of aIC➔NAcc terminals suppressed number of press bouts in the context test (*n* = 7–8 mice per group; two‐way ANOVA: group effect: *F*_(1, 13)_=16.82, *p* = 0.00012, time effect: *F*_(1, 13)_= 3.392, *p* = 0.0885, interaction: *F*_(1, 13)_= 21.20, *p* = 0.0005, Sidak's post hoc test, eYPF: *p* = 0.0016, context test: *p* < 0.0001). (N) Experimental timeline for cue reinstatement. (O–P) Inhibition of aIC➔NAcc terminals suppressed active lever pressing during cue reinstatement. (*n* = 6–7 mice per group; O: two‐way ANOVA, group effect: *F*_(1, 11)_=9.860, *p* = 0.0094, time effect: *F*_(1.432, 15.75)_ = 20.36, *p* = 0.0001, interaction: *F*_(1.432, 15.75)_ = 5.177, *p* = 0.0207; P: two‐way ANOVA, group effect: *F*_(1, 11)_=6.048, *p* = 0.0317, time effect: *F*_(1, 11)_= 24.88, *p* = 0.0004, interaction: *F*_(1, 11)_= 3.3231, *p* = 0.0957, Sidak's post hoc test, eYFP: *p* = 0.0016, cue rein: *p* = 0.0238). (Q) Cumulative frequency distribution of interpress intervals from eYFP and eNpHR3.0 mice during cue rein show similar interpress intervals for eYFP and eNpHR3.0 groups. Inset shows box and whisker plots for interpress intervals during cue rein. (R) Inhibition of aIC➔NAcc terminals suppressed number of press bouts during cue rein (*n* = 6–7 mice per group; two‐way ANOVA: group effect: *F*_(1, 15)_=5.750, *p* = 0.0354, time effect: *F*_(1, 11)_= 26.01, *p* = 0.0003, interaction: *F*_(1, 11)_= 4.880, *p* = 0.0493, Sidak's post hoc test, eYFP: *p* = 0.0009, cue test: *p* = 0.0144). Data are mean ± SEM *p < 0.05, **p < 0.01, ***p < 0.001, ****p < 0.0001. aIC, anterior insular cortex; IPI, interpress interval; NAcc, nucleus accumbens core; rein, reinstatement; SA, self‐administration. Data are mean ± SEM. *p < 0.05, **p < 0.01, ***p < 0.001, ****p < 0.0001. aIC, anterior insular cortex; contra, contralateral: ipsi, ipsilateral; NAcc, nucleus accumbens core.

To determine whether aIC➔NAcc neurons drive opioid seeking we employed pathway‐specific optogenetics to selectively silence aIC➔NAcc terminals during opioid‐seeking behaviour. First, we targeted the aIC using a virus encoding for the inhibitory opsin halorhodopsin (AAV5‐CaMKIIα‐eNpHR3.0‐eYFP) or eYFP (AAV5‐CaMKIIα‐eYFP) and implanted optical fibres above the lateral NAcc (Figure 2A–C). We leveraged our previously established head‐fixed heroin self‐administration paradigm [18] (Figure 2D), which allows for precise measurement of operant drug‐seeking behaviour concurrent with optogenetics. Animals were trained to press an active lever for delivery of a tone that preceded an intravenous infusion of heroin and a 20‐s timeout period (Figure 2E). Animals learned to discriminate between the active and inactive lever and robustly increased active lever presses across 14 days of heroin self‐administration (Figure 2F). Moreover, the interpress interval decreased from early (days 1–2) to late (days 13–14) heroin self‐administration (Figure 2G), and both the number of infusion bouts and press bouts escalated across heroin self‐administration (Figure 2H). Hence, we observed a robust increase in operant responding for heroin across the self‐administration period.

Next, we considered a role for aIC➔NAcc neurons in mediating context‐associated opioid seeking following short‐term abstinence, as previous studies support a role for the aIC and aIC➔NAcc pathway in morphine CPP [10, 15]. After the last day of heroin self‐administration, animals underwent 72 h of forced abstinence in the home cage before being returned to the behavioural chamber for the context test (Figure 2I), wherein an active lever press no longer resulted in the presentation of cue or delivery of drug. Optogenetic inhibition of the aIC➔NAcc terminals markedly suppressed the total number of active lever presses during the context test (Figure 2J,K) and the number of active presses during the laser on and laser off epochs (Figure S2A). In a small group of animals, we tested whether the aIC to medial NAcc projection neurons influenced opioid seeking behaviour but consistent with our tracing data that show the limited aIC projections to this region, we did not detect a difference in lever pressing compared to the eYFP controls (Figure S2B). Compared to eYFP animals, eNpHR3.0 animals displayed increased interpress intervals during the context test (Figure 2L). Finally, while eYFP animals displayed an increased number of press bouts compared to baseline (final day of self‐administration), total press bouts in the eNpHR3.0 group were not different to baseline and were significantly suppressed compared to the eYFP group during the context test (Figure 2M). Together, these results reveal that following an acute abstinence period aIC➔NAcc neurons are recruited by drug‐associated contexts to drive opioid seeking.

Whether aIC➔NAcc neurons are also required for cued opioid seeking following opioid self‐administration has never been tested. Therefore, we used an extinction‐reinstatement paradigm to determine the necessity of this output for cued heroin seeking. Animals underwent extinction training for a minimum of 10 days before the cue‐reinstatement test (Figure 2N), in which an active lever press resulted in presentation of the heroin‐associated cue. Optogenetic inhibition of aIC➔NAcc terminals resulted in a small but significant decrease in active lever presses across the cue reinstatement test (Figure 2O). While the eYFP group displayed increased active lever presses during the cue reinstatement test compared to the last day of extinction, active lever presses in the eNpHR3.0 group were not different from extinction and were markedly reduced compared to the eYFP group (Figure 2P). We observed a main effect of group when analysing the active lever presses during the laser on and laser off epochs (Figure S2C). However, unlike in the context test, there were no differences in the cumulative distribution of interpress intervals between the eYFP and eNpHR3.0 groups during the cue reinstatement test (Figure 2Q). Lastly, while the eYFP group increased the number of press bouts compared to the last day of extinction, total press bouts in the eNpHR3.0 group were not different from extinction and were suppressed compared to the eYFP group during the cue‐reinstatement test (Figure 2R). Our results indicate that aIC➔NAcc neurons are also required for cued opioid seeking following extinction training. Altogether, these data reveal that aIC➔NAcc neurons are engaged by external opioid‐associated cues to promote opioid‐seeking behaviour.

### 2.3 Optogenetic Inhibition of aIC➔ NAcc Terminals Does Not Alter Sucrose Seeking

**FIGURE 3:**
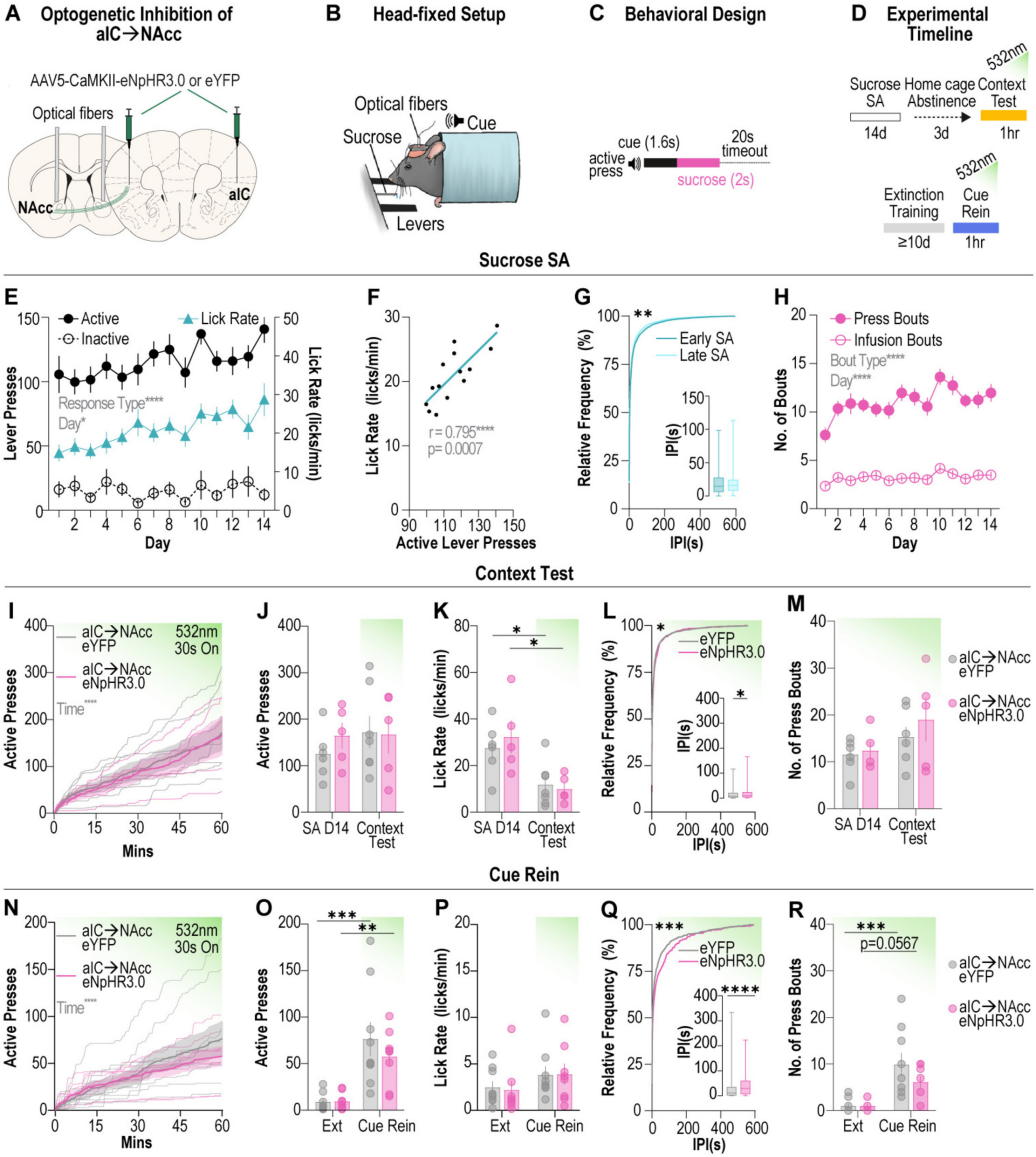
aIC➔NAcc inhibition has no effect on sucrose seeking. (A) Viral approach used to optogenetically inhibit aIC➔NAcc terminals. (B) Schematic showing head‐fixed behavioural apparatus. (C) Behavioural paradigm for head‐fixed sucrose self‐administration. (D) Experimental timeline for context test (top) and cue reinstatement (bottom). (E) Grouped data for active and inactive lever presses and mean lick rate across acquisition of sucrose self‐administration. (*n* = 29 mice; mixed‐effects model, response type effect: *F*_(2,84)_ = 201.8, *p* < 0.0001, day effect: *F*_(9.565794.7)_ = 2.232, *p* = 0.0161, interaction: *F*_(19.13794.7)_ = 1.135, *p* = 0.3092). (F) Correlation between mean lick rate and mean active lever press for each day of sucrose self‐administration (*n* = 29 mice across 14 days; Pearson's correlation, *r* = 0.7947, *p* = 0.0007). (G) Cumulative frequency distribution of interpress intervals from early (days 1–2) and late (days 13–14) sucrose self‐administration (*n* = 29 mice; Kolmogorov–Smirnov test, Kolmogorov–Smirnov *D* = 0.1217, *p* = 0.0033). Inset shows box and whisker plot for interpress intervals from early and late sessions. (H) Infusion bouts, defined as three or more infusions in a 2‐min period, and press bouts, defined as three or more lever presses with a 25‐s period, increased across sucrose self‐administration. (*n* = 29 mice; two‐way ANOVA, bout‐type effect: *F*_1,56_ = 392.1, *p* < 0.0001, day effect: *F*_(8.547478.6)_ = 4.087, *p* < 0.0001, interaction: *F*_(8.547478.6)_ = 1.466, *p* = 0.1619). (I–J). Inhibition of aIC➔NAcc terminals has no effect on active lever pressing during the context test (*n* = 5–7 mice per group; I: time effect: *F*_(1.346,13.46)_ = 30.75, *p* < 0.0001). (K) Mean lick rate during context test did not different between eYFP and eNpHR3.0 groups (*n* = 5–7 mice per group; two‐way ANOVA, day effect: *F*_(1, 9)_=19.47, *p* = 0.0017, Sidak's post hoc test, eYFP: *p* = 0.0457, eNpHR3.0: *p* = 0.0139). (L) Cumulative frequency distribution of interpress intervals from eYFP and eNpHR3.0 mice during the context test (*n* = 5–7 mice per group; Kolmogorov–Smirnov test, Kolmogorov–Smirnov *D* = 0.1167, *p* = 0.0429). Inset shows box and whisker plot for interpress intervals during context test (*n* = 5–7 mice per group, Mann–Whitney test, Mann–Whitney *U* = 35 988, *p* = 0.0231). (M) Inhibition of aIC➔NAcc terminals had no effect on number of press bouts in the context test. (N–O) Inhibition of aIC➔NAcc terminals had no effect on active lever pressing during cue reinstatement (*n* = 8–9 mice per group; N: two‐way ANOVA, time effect: *F*_(1.249, 18.73)_ = 31.49, *p* < 0.0001; O: two‐way ANOVA, day effect: *F*_(1, 15)_=29.60, *p* < 0.0001, Sidak's post hoc test, eYFP: *p* = 0.0013, eNpHR3.0: *p* = 0.0282). (P) Mean lick rate during cue rein did not different between eYFP and eNpHR3.0 groups. (Q) Cumulative frequency distribution of interpress intervals from eYFP and eNpHR3.0 mice during cue rein (*n* = 8–9 mice per group; Kolmogorov–Smirnov test, Kolmogorov–Smirnov *D* = 0.2569, *p* = 0.0001). Inset shows box and whisker plot for interpress intervals during cue rein (*n* = 8–9 mice per group, Mann–Whitney test, Mann–Whitney *U* = 9196, *p* < 0.0001). (R) Inhibition of aIC➔NAcc terminals had no effect on number of press bouts in the context test. (*n* = 8–9 mice per group; two‐way ANOVA, day effect: *F*_(1, 15)_=23.11, *p* = 0.0002, Sidak's post hoc test, eYFP: *p* = 0.0009, eNpHR3.0: *p* = 0.00569). Data are mean ± SEM *p < 0.05, **p < 0.01, ***p < 0.001, ****p < 0.0001. aIC, anterior insular cortex; rein, reinstatement; self‐administration, self‐administration.

To determine whether manipulation of the aIC➔NAcc projection also impacts natural reward seeking, we combined pathway‐specific optogenetics with our previously established head‐fixed sucrose self‐administration paradigm [18] (Figure 3A,B). In this paradigm, an active lever press results in presentation of a tone followed by delivery of a liquid sucrose reward (12.5 μL of 12.5% sucrose) and a 20‐s timeout period (Figure 3C), designed to match the heroin self‐administration paradigm. We then tested animals following sucrose self‐administration in the context test and the cue reinstatement test under the same conditions described for heroin self‐administration (Figure 3D). Across self‐administration, animals learned to press the active lever for delivery of a sucrose reward (Figure 3E). We observed that lick rate correlated strongly with active lever presses across heroin self‐administration (Figure 3F). The interpress interval differed from early (days 1–2) to late (days 13–14) sucrose self‐administration (Figure 3G), while the number of infusion and press bouts increased across sucrose self‐administration (Figure 3H), demonstrating robust operant responding for sucrose reward.

During the context test, optogenetic inhibition of aIC➔NAcc terminals had no effect on total active lever pressing (Figure 3I,J) or lever pressing during laser on and laser off epochs between eYFP and eNpHR3.0 groups, although a small but significant interaction between group and laser status was observed (Figure S3A). Lick rate decreased compared to baseline in both groups likely due to the omission of cue and sucrose during this test. Importantly, there was no difference in lick rate between eNpHR3.0 and eYFP groups during the context test (Figure 3K). We observed a slight difference in interpress interval in the eNpHR3.0 group compared to the eYFP group (Figure 3L) but did not find any differences in press bouts between groups during the context test (Figure 3M). Together, these results demonstrate that inhibition of aIC➔NAcc neurons has no effect on context‐associated natural reward seeking.

Following a minimum of 10 days of extinction training, animals underwent a cue reinstatement test with optogenetic inhibition of aIC➔NAcc terminals. There was no difference in active lever pressing between eNpHR3.0 and eYFP during cue reinstatement, with both groups increasing total lever presses compared to the last day of extinction (Figure 3N,O), and no differences in active presses during laser on and off epochs (Figure S3B). Lick rate did not differ between groups or between days (Figure 3P). We did observe a significant difference in interpress interval in the eNpHR3.0 group compared to eYFP (Figure 3Q). However, there were no differences in the number of press bouts between groups, with total press bouts increasing in the eYFP group from extinction and a trend towards a significant increase in the eNpHR3.0 group (Figure 3R). Therefore, these results indicate that inhibition of aIC➔NAcc neurons does not alter cue‐induced sucrose seeking. Altogether, our data reveal that aIC➔NAcc neurons are selectively required for motivated opioid seeking and not natural reward seeking, supporting a role for this projection as key mediator of relapse to opioid seeking.

## 3 Discussion

Our data reveal that aIC➔NAcc projection neurons are critical drivers of opioid‐seeking behaviour, with aIC➔NAcc projection neurons activated by opioid‐associated contexts and opioid‐paired cues to selectively drive opioid‐seeking behaviour. Importantly, we observed no effect of manipulation of this pathway on natural reward seeking under the same conditions, suggesting the aIC➔NAcc pathway may undergo neurobiological adaptation specifically in response to drug self‐administration. This is the first time aIC➔NAcc neurons have been investigated using a preclinical opioid self‐administration and reinstatement paradigm, an experimental approach that has strong predictive validity in determining effective medications for the treatment of opioid abuse [19, 20]. Therefore, our results highlight the potential of modulation strategies that target aIC➔NAcc neurons in the treatment of OUD and prevention of relapse.

Consistent with our results, inhibition of aIC➔NAcc neurons suppresses reinstatement of morphine CPP [15], further supporting a role for this projection as a key mediator of relapse. However, this same study found no effect of aIC➔NAcc inhibition on the expression of morphine CPP before extinction training [15]. In contrast, our results demonstrate that aIC➔NAcc neurons are recruited during opioid seeking regardless of extinction training. This disagreement likely reflects the fundamental differences between self‐administration and CPP paradigms, as drug seeking following noncontingent drug delivery is known to engage different neural mechanisms [21].

Our results demonstrate that the aIC projects primarily to the lateral NAcc, not the medial NAcc, and that only inhibition of the aIC to lateral NAcc projection disrupts heroin‐seeking behaviour in the context test. This suggests a functional difference between these two projections that is possibly explained by the greater number of aIC cells projecting to lateral NAcc core. Future studies aim to determine whether there are separate populations of aIC neurons projecting to the lateral versus medial NAcc core and whether these circuits differentially regulate other reward‐related behaviours.

Of the limited studies that have used opioid self‐administration paradigms to investigate a role for the aIC in opioid seeking, results are conflicting. Some studies find that global silencing of the aIC either through reversible pharmacological inhibition or excitotoxic lesions enhances cued‐heroin seeking following heroin self‐administration [6, 7], suggesting that the aIC exerts inhibitory control over opioid‐seeking behaviour. Conversely, others report that inhibiting the aIC suppresses fentanyl seeking following fentanyl self‐administration and voluntary abstinence, supporting a role for the aIC as a driver of relapse to opioid seeking [8, 9]. While differences in animal models (rats vs. mice), behavioural paradigm (extinction vs. voluntary/forced abstinence) or even type of opioid administered could explain the discrepancies in results here, the nonspecific nature of pharmacological inhibition or lesion studies is likely a key factor underlying the conflicting findings, especially in a region with such anatomical complexity as the aIC. Anatomical tracing studies describe the inputs to this region as diverse and nonorganized, with pockets of the aIC receiving inputs from one region or sending projections to another [3, 4]. Thus, studies indiscriminately silencing the aIC are inherently difficult to interpret and compare to projection‐specific approaches due to the heterogeneity of this region.

More broadly, our results align with the literature examining the role of aIC output circuits in drug seeking behaviour following exposure to drugs from other classes. The aIC➔NAcc pathway appears to be relevant to alcohol use disorder, where inhibition has been observed to suppress alcohol self‐administration [22, 23], and aversion resistant alcohol consumption [11]. The aIC also sends largely nonoverlapping projections to the amygdala [24], where the aIC to central amygdala (CeA) circuit has been found to regulate relapse to methamphetamine seeking following voluntary abstinence [12]. While less prominent, the aIC sends projections to midbrain and hindbrain regions, with the aIC to ventral tegmental area (VTA) neurons required for amphetamine CPP [13], and the aIC to locus coeruleus (LC) neurons necessary for aversion‐resistant alcohol consumption [14]. Together, these results highlight the aIC as a central node engaged by drugs of abuse to drive maladaptive drug‐seeking behaviours and promote relapse in response to external triggers. Other excitatory cortical inputs into the NAc are understood to be required for opioid seeking in response to external drug‐associated stimuli. For example, the ventromedial prefrontal to NAc shell pathway appears to be required for context‐induced heroin seeking following extinction training [25], while the dorsomedial prefrontal to NAc core pathway is required for cue‐induced heroin seeking following extinction training [16, 17]. Potentially, aIC inputs to the NAc overlap with inputs from other cortical regions and activate similar populations of downstream neurons. Within the NAc, dopamine‐1‐like receptor (Drd1) expressing neurons in the lateral NAc shell are required for context‐induced heroin seeking, while Drd1 neurons in the NAc core are required for discrete drug‐associated cues to drive heroin seeking [26]. Future work should consider the downstream targets of the aIC within the NAc and how the aIC projections might interact with other NAc inputs.

Notably, the IC is considered a key substrate in interoception, the process by which bodily signals are detected by the brain. Interoception is involved in multiple aspects of drug abuse from the pleasurable sensations associated with drug use to the psychophysiological withdrawal symptoms that precede drug seeking [27, 28, 29, 30]. This established role for the IC in processing internal signals raises questions as to how a specialized interoceptive region comes to respond to external drug‐associated stimuli, and what distinguishes IC cue reactivity from that observed in other cortical areas such as the prefrontal or orbitofrontal regions. One hypothesis suggests that acute interoceptive signals related to drug use are gradually associated with external drug‐associated stimuli through dopamine‐dependent neuroplasticity within the IC and connected brain regions [29]. This results in a persistent representation of drug‐related internal state within the IC that is recalled upon exposure to external cues, thereby triggering conscious urges to seek drugs [29]. While this has never been directly tested, there is now evidence that food‐paired cues transiently evoke satiety‐like neuronal activity patterns in the IC [31], supporting the idea that external reward‐paired cues act on the IC to recall learned physiological associations. Future studies utilizing technological advances in neuroscience, such as single‐cell calcium imaging, will be critical in addressing unanswered questions, such as whether external drug‐associated stimuli engage the same neurons as drug‐related interoceptive signals and determining the cell‐type and circuit‐specific mechanisms through which the IC promotes drug seeking. A recent review of the role of the IC in addiction highlights orexin receptors and Drd1 and Drd2 receptors in the aIC as potential mechanisms underlying nicotine‐ and cocaine‐seeking behaviour; however, whether this is also the case in opioid seeking is not yet known [32].

The present study has several limitations. Firstly, while we observed no obvious differences between males and females, the study was not sufficiently powered to assess minor sex differences. In addition, in the heroin experiments, optogenetic inhibition during the context test could have led to a persistent reduction in seeking behaviour in the cue reinstatement test, although we did not detect differences in lever presses during extinction between the two groups, suggesting that the optogenetics protocol only acutely disrupted behaviour. We should also note that while we examined behaviour in response to the heroin‐associated context following short term abstinence, we did not use the established ABA model of context‐induced reinstatement [33]; therefore, it is important that future studies confirm the involvement of the aIC➔NAcc neurons in context‐induced reinstatement. Additionally, we only tested whether the aIC➔NAcc pathway is required for opioid seeking following forced abstinence at one time point (3 days after heroin self‐administration). Thus, it remains to be established if this circuit is necessary for seeking acutely following heroin self‐administration and following protracted periods of abstinence (> 3 days). In this study, while we show that aIC➔NAcc neurons are necessary for heroin seeking, we did not perform a sufficiency experiment. Future experiments could determine whether activation of these neurons is sufficient to drive seeking in the absence of external cues. Finally, we used a head‐fixed heroin self‐administration paradigm, which may add additional stress to the animals throughout the experimental period. However, we have previously validated that other circuits critical to relapse behaviour are necessary in our head‐fixed paradigm [17], suggesting that this paradigm recruits the same circuits as freely moving self‐administration paradigms. This head‐fixed paradigm also offers improved control during tethered optogenetics experiments and is compatible with high‐resolution imaging techniques such as two‐photon calcium imaging, which would enable longitudinal tracking of individual IC neurons across drug self‐administration to relapse.

In conclusion, we find a role for aIC➔NAcc neurons in promoting relapse to opioid seeking in response to external cues. This role is specific to drug seeking, as manipulation of aIC➔NAcc neurons during natural reward seeking had no effect, highlighting the potential of aIC➔NAcc neurons as a target in novel treatments for substance use disorders. Future experiments should aim to determine the cell‐type specific aIC➔NAcc mechanisms underlying drug seeking and how external and internal signals are integrated in the IC to guide motivated behaviour.

## 4 Materials and Methods

### 4.1 Animals

All experiments were approved by the Institutional Animal Care and Use Committee (IACUC) at the Medical University of South Carolina in accordance with the NIH‐adopted Guide for the Care and Use of Laboratory Animals. Adult male and female Ai‐14 transgenic mice or wild‐type mice on a C57BL6/J background were group‐housed preoperatively and single‐housed postoperatively, with access to standard chow and water ad libitum throughout all experiments. Mice were at least 8 weeks of age and a minimum of 18.5 g at study onset. Male and female mice were randomly assigned to experimental groups. Mice were housed under a reverse 12‐h light cycle (lights off at 8:00 AM), with experiments performed during the dark phase.

### 4.2 Surgery

For intracranial or intravenous catheter surgeries, mice were anaesthetised with isoflurane (1%–2.5% in oxygen; 1 L/min). Ophthalmic ointment (Akorn), topical anaesthetic (2% Lidocaine; Akorn) and analgesic (Carprofen, 20 mg/kg, subcutaneous injection) were given preoperatively and intraoperatively for health and pain management. An antibiotic (Cefazolin, 200 mg/kg, subcutaneous injection) was given postoperatively to reduce the possibility of infection. For intracranial surgery, anaesthetised mice were placed within a stereotactic frame (Kopf Instruments).

To quantify aIC➔NAcc projection neurons, retrogradely trafficked virus encoding for Cre‐recombinase (rgAAV2‐CAG‐Cre, UNC; 400 nL/injection site, unilateral) was infused into the lateral NAcc (AP: +1.25 mm; ML: +1.34 mm; DV: −4.55 mm: relative to bregma) or the medial NAcc (AP: +1.25 mm; ML: +0.8 mm; DV: −4.55 mm). Viral placements were confirmed post mortem via histology (S1A&B).

For optogenetic manipulation of aIC➔NAcc neurons, we infused a virus encoding for one of two constructs (AAV5‐CaMKIIα‐eNpHR3.0‐eYFP or AAV5‐CaMKIIα‐Eyfp, UNC Vector Core; 300 nL/injection site) into the aIC (AP: +1.75 mm; ML: ±3.1 mm; DV: −3.75; and AP: +1.60 mm; ML: ±3.3 mm; DV: −3.75). Custom‐made optical fibres [34] were implanted dorsal to the lateral NAcc (AP: +1.25 mm; ML: ±0.95 mm; DV: −4.40 mm) or medial NAcc (AP: +1.25 mm; ML: ±0.90 mm; DV: −4.40 mm), allowing laser‐evoked inhibition of aIC➔NAc terminals. A stainless‐steel head ring was cemented around the optical fibre using dental cement and skull screws. Optical fibre and viral placements were confirmed post mortem via histology (Figures S2D and S3C).

Mice assigned to heroin self‐administration underwent 7 days of recovery from intracranial surgery before catheterization occurred. Mice were implanted with custom‐made intravenous catheters using a method previously described [18]. Following 5 days of recovery, mice began behavioural experiments wherein catheters were flushed daily with heparinized saline (60 units/mL, 0.04 mL) to maintain patency. Mice with nonpatent catheters were excluded from the study.

### 4.3 Head‐Fixed Behaviour

Experiments involving heroin or sucrose self‐administration were performed based on a previous study wherein we developed a model of natural‐ and drug‐reward seeking in head‐restrained mice [18]. After recovery from surgery, mice were habituated for 2 days to the head‐fixed apparatus and behavioural chamber during 45‐min sessions where levers were not presented.

### 4.4 Head‐Fixed Heroin Self‐Administration

Mice (*n* = 15, 5F, 10M) underwent 14 days of heroin self‐administration, during which two levers were placed in front of the animal within forelimb reach. Pressing the active lever, but not the inactive lever, resulted in the presentation of a tone cue (8 kHz, 1.6 s) followed immediately by the intravenous infusion of heroin (2 s). A timeout period (20 s) was given after each reinforced active lever press, wherein active lever pressing had no effect. Mice were trained on a fixed‐ratio 1 (FR1) schedule of reinforcement using a decreasing dose design (days 1–2: 0.1 mg/kg/12.5 μL heroin, 10 infusion maximum; days 3–4: 0.05 mg/kg/12.5 μL heroin, 20 infusion maximum; days 5–14: 0.025 mg/kg/12.5 μL heroin, 40 infusion maximum), for a total of 1 mg/kg of heroin per 1‐h session.

Following 72 h of forced abstinence in the home cage, mice were returned to the behaviour chamber for the context test, wherein active lever presses resulted in neither cue nor drug delivery (extinction conditions). For both eNpHR3.0 or control eYFP mice, the laser (532 nm; 7.5‐11 mW per fibre) was displayed (constant light) for 30‐s intervals once/min throughout the session.

Following the context test, mice underwent 1‐h extinction training sessions until extinction criteria were reached. Extinction criteria were determined a priori, as (1) ≥ 10 days of extinction training and (2) the last 2 days of extinction training resulting in ≤ 20% of the average active lever pressing observed during the last 2 days of acquisition. Once extinction criteria were achieved, mice underwent a 1‐h cue‐reinstatement test, where active lever presses resulted in cue presentation as in acquisition; however, drug infusions were omitted. A timeout period (20 s) was given after the onset of each cue, wherein active lever pressing did not result in cue delivery. Laser settings were the same as described above.

### 4.5 Head‐Fixed Sucrose Self‐Administration

A separate cohort of mice (*n* = 29, 11F, 18M) were assigned to 14 days of sucrose self‐administration, during which two levers were placed in front of the animal within forelimb reach, and a lick spout was adjusted to the height of the animal's mouth. Pressing the active lever, but not the inactive lever, resulted in the presentation of a tone cue (8 kHz, 1.6 s) followed immediately by an infusion of sucrose (12.5% in tap water, 2‐s epoch). A timeout period (20 s) was given after each reinforced active lever press, wherein active lever pressing had no effect. Mice were trained on a fixed‐ratio 1 (FR1) schedule of reinforcement and were capped at 40 sucrose infusions or 1 h, to mimic the heroin self‐administration paradigm.

A subset of mice that completed sucrose self‐administration (*n* = 12, 5F, 7M) were given 72 h of home cage abstinence before returning to the behaviour chamber and tested for the context test, wherein active lever presses resulted in neither cue nor sucrose delivery. Optogenetic manipulations were performed as described above.

A separate group of mice (*n* = 17, 6F, 11M) in the sucrose group underwent 1‐h extinction training sessions following the last day of sucrose self‐administration until extinction criteria (described above) were reached. Mice then underwent a 1‐h cue‐induced reinstatement, where active lever presses resulted in cue presentation as in acquisition; however, sucrose infusions were omitted. Optogenetic manipulations were performed as described under heroin self‐administration.

### 4.6 Histology and Immunohistochemistry

Following the experimental end point, mice were transcardially perfused with 4% paraformaldehyde (PFA) in phosphate buffered saline (PBS). Brains were stored at 4°C in PFA for 24 h before being sliced at 50 μm on a vibratome in 0.1 M PBS and stored in 0.1 M PBS with 1% sodium azide at 4°C*.* Prior to imaging, tissue sections were mounted on premium frosted microscope slides (Fisherbrand) with DAPI fluromount‐G (Southern Biotech).

Ai‐14 mice were perfused 5–6 weeks following intracranial surgery. Brains were extracted immediately and submerged in PFA for 24 h before slicing. Brain sections were imaged at 10× as a z‐series (1024 × 1024 frame size) using a Leica SP8 laser‐scanning confocal microscope with a 10× air objective and an optical section thickness of 1 μm. For detection of Tdtomato+ cells, an OPSL 552‐nm laser line was used.

Mice from the heroin or sucrose self‐administration experiments were perfused at humane endpoint or following final test in behavioural paradigm. The brain was left in the skull with head‐cap intact and submerged for 72 h in PFA before being extracted to allow for clear visualization of the optical fibre placement. Following slicing, free floating tissue sections were blocked in 0.1 PBS with 2% Triton X‐100 (PBST) with 2% normal goat serum (NGS, Jackson Immuno Research) for 2 h at room temperature with agitation. Sections were then incubated overnight at 4°C with agitation in GFP primary antibody (chicken polyclonal anti‐GFP antibody, Abcam) diluted in 2% PBST with 2% NGS, washed 3 times for 5 min in PBST, then incubated in the appropriate secondary antisera diluted in PBST with 2% NGS for 4 h at room temperature with agitation. Secondary antibodies (goat anti‐chicken Alexa Fluor 488, Invitrogen) were used at a concentration of 1:1000. Sections were then washed 3 times for 5 min in PBS before mounting and imaging. Brain sections were imaged on a Leica THUNDER Imager Tissue for confirmation of viral expression and optical fibre placement. For representative images, sections were imaged using a Leica SP8 laser‐scanning confocal microscope, using an OPSL 488 nm laser line to detect eYPF+ cells.

### 4.7 Quantification and Statistical Analysis of Retrograde Tracing Data

Ipsilateral and contralateral regions from 12 aIC sections from three animals with lateral NAcc injections and 12 aIC sections from two animals with medial NAcc injections were analysed using Imaris 9.0 (Bitplane). Within Imaris, the ‘spots’ function was used to calculate the number of Tdtomato+ neurons, and this was normalized to the 3D volume of tissue determined by Imaris. A two‐way ANOVA was used to compare the number of aIC projection neurons based on hemisphere (ipsilateral vs. contralateral) and injection site (lateral vs. medial) with a Sidak's post hoc comparison. Tracing data are represented as mean ± standard error of the mean. All statistical analyses were performed using Prism 10.0 (GraphPad Prism) statistical software.

### 4.8 Statistical Analysis of Behavioural Data

Behavioural data for each animal from each session was analysed using a custom written python script and Prism 10.0 (GraphPad Prism). To determine the press bout interval, we used the median interpress interval (24.7 s) during an infusion bout during heroin self‐administration (all animals, all days, *n* = 210 sessions, *n* = 493 bouts). Therefore, we considered three or more active lever presses within a 24.7‐s period to be one press bout and used this to measure the number of press bouts for both heroin and sucrose self‐administration groups. For optogenetics tests, we analysed both the full‐hour session including both laser on and laser off epochs (Figures 2 and 3), and the active lever presses during laser on and laser off epochs separately (Figures S2A, S2C, S3A and S3B). For sucrose self‐administration, licking data were filtered (minimum of 110‐ms interval between licks) to exclude instances where the animal holds the lick spout, and days where sucrose was administered and less than 10 licks were recorded were excluded (*n* = 1 eYFP animal during SAD14). For cumulative frequency distribution, a Kolmogorov–Smirnov test was used to determine differences in distribution between conditions or groups, and a Mann–Whitney test was used to compare the medians between conditions or groups. For correlating lick rate with active lever presses, a Pearson's correlation was used. For comparing inhibition of the aIC to medial NAcc to eYFP and aIC to lateral NAcc on context‐associated heroin seeking, a one‐way ANOVA with Tukey's post hoc test was used. For all other behaviour data, a two‐way ANOVA or mixed‐effects models was performed with Sidak's post hoc test where applicable.

## Author Contributions

Experimental design: REC, MDS and JMO. Data collection: REC, BEP, IED, JEP, ACT, AMW, JB. Analysis and interpretation of data: REC and JMO. Statistical analysis: REC. Drafting of manuscript: REC. Critical revision of the manuscript: REC, MDS and JMO. Obtained funding: JMO.

## Funding

This study was supported by grants from the National Institute on Drug Abuse (NIH/NIDA; R01‐DA062041, R01‐DA054271, R01‐DA051650) and the United States Department of Veterans Affairs, Biomedical Laboratory Research and Development Service (VA BLR&D; I01‐BX006179).

## Ethics Statement

All experiments were approved by the Institutional Animal Care and Use Committee (IACUC) at the Medical University of South Carolina in accordance with the NIH‐adopted Guide for the Care and Use of Laboratory Animals.

## Conflicts of Interest

The authors declare no conflicts of interest.

## Supporting information


**Table S1:** Supporting Information.

**Figure S1:** adb_70118‐sup‐0002‐Supplement.docx. **Injection site mapping for Ai‐14 tracing study.** (**A**) Placements for lateral NAcc injection sites (*n* = 3). (**B**) Placements for medial NAcc injection sites (*n* = 2). NAcc, nucleus accumbens core.
**Figure S2: Supplemental data for heroin self‐administration experiment.** (**A**) Active presses during both laser on and laser off epochs were significantly higher in the eYFP group compared to the eNpHR3.0 group during the context test (*n* = 7–8 mice per group; mixed‐effects model, group effect: *F*_(1, 13)_ = 24.51, *p* = 0.0003, laser effect: *F*_(1, 13)_ = 0.3883, *p* = 0.5440, interaction: *F*_(1, 13)_ = 0.3023, *p* = 0.5917, Sidak's post hoc test, laser on: *p* = 0.0001, laser off: *p* = 0.0002). (**B**) Total active lever presses during context test did not differ compared to eYFP when optical fibres were targeted to the medial NAcc and were significantly higher compared to when the aIC➔lateral NAcc pathway was targeted (Data for eYFP and lateral NAcc eNpHR3.0 also appear in Figure 2K; *n* = 4–8, one‐way ANOVA, *F*_(2, 16)_=9.337, *p* = 0.0021, Tukey's post hoc test, eYFP versus Med eNpHR3.0: *p* = 0.9706 eYFP versus Lat eNpHR3.0: *p* = 0.0036, Med eNpHR3.0 versus Lat eNpHR3.0: *p* = 0.0092). (**C**) Active presses during laser on and laser off epochs were higher in the eYFP group compared to the eNpHR3.0 group during cue reinstatement (*n* = 6–7 per group, two‐way ANOVA, group effect: *F*_(1, 11)_ = 4.861, *p* = 0.0497, laser effect: *F*_(1, 11)_ = 0.2940, *p* = 0.5985, interaction: *F*_(1, 11)_ = 1.176, *p* = 0.3014). (**D**) Viral expression of eYFP and eNpHR3.0‐eYFP (top) and lateral (purple) or medial (red) optical fibre placement (bottom) for animals in heroin self‐administration paradigm (*n* = 17 lateral NAcc, *n* = 4 medial NAcc). Data are mean ± SEM **p < 0.01; ***p < 0.001. Lat, lateral; Med, medial; NAcc, nucleus accumbens core.
**Figure S3: Supplemental data for sucrose self‐administration experiment. (A**) There was a small but significant interaction between laser status and experimental group in active lever presses during the context test (*n* = 5–7, mixed‐effects analysis, group: *F*_(1, 10)_ = 0.009376, *p* = 0.9248, laser status: *F*_(1, 10)_ = 0.07256, *p* = 0.7931, interaction: *F*_(1, 10)_ = 6.278, *p* = 0.0311). (**B**) Active presses during laser on and laser off epochs did not differ between eYFP and eNpHR3.0 groups during cue reinstatement. (**C**) Viral expression of eYFP and eNpHR3.0‐eYFP (top) and optical fibre placement (bottom) for animals in sucrose self‐administration paradigm (*n* = 29). NAcc, nucleus accumbens core.

## Data Availability

Data are available upon request from the corresponding authors.
