# Supplementary material for: Activation of Insula‐Accumbal Projection Neurons Is Required for Relapse‐Like Behaviour Following Opioid Self‐Administration
Source: Addict Biol. 2026 Jan 12;31(1):e70118. doi: 10.1111/adb.70118 (PMC12795779; doi:10.1111/adb.70118)
Supplement: Supplementary file 1 — Table S1: Supporting Information. [file ADB-31-e70118-s001.pdf]

Figure	Statisitical test	n	*p* -value	Post test	*p* -value
1G	Two-way ANOVA	Med NAcc=12, Lat NAcc=12		Ipsi:Med NAcc vs. Contra:Med NAcc	0.6636
	Injection Site Effect: F (1, 44) = 100.5		<0.0001	Ipsi:Lat NAcc vs. Contra:Lat NAcc	<0.0001
	Hemisphere Effect: F (1, 44) = 22.62		<0.0001	Ipsi:Med NAcc vs. Ipsi:Lat NAcc	<0.0001
	Interaction: F (1, 44) = 9.399		0.0037	Contra:Med NAcc vs. Contra:Lat NAcc	<0.0001
2F	Mixed-effects Model	n=15			
	Lever Effect: F (1, 28) = 104.4		<0.0001		
	Day Effect: F (6.423, 178.8) = 12.62		<0.0001		
	Interaction: F (6.423, 178.8) = 8.209		<0.0001		
2G (main)	Kolmogorov-Smirnov test	Early Acq=772, Late Acq=5661			
	Kolmogorov-Smirnov D= 0.3632		<0.0001		
2G (inset)	Mann Whitney Test	Early Acq=772, Late Acq=5661			
	Mann-Whitney U = 16880		<0.0001		
2H	Mixed-effects Model	n=15			
	Bout Type Effect: F (1, 28) = 144.4		<0.0001		
	Day Effect: F (6.939, 193.2) = 17.27		<0.0001		
	Interaction: F (6.939, 193.2) = 5.317		<0.0001		
2J	Two-way ANOVA	eYFP=8, eNpHR3.0=7			
	Group Effect: F (1, 13) = 36.89		<0.0001		
	Time Effect: F (1.418, 18.43) = 33.49		<0.0001		
	Interaction: F (1.418, 18.43) = 18.93		<0.0001		
2K	Two-way ANOVA	eYFP=8, eNpHR3.0=7		SAD14: eYFP vs Context: eYFP	0.4395
	Group Effect: F (1, 13) = 30.34		0.0001	SAD14: eNpHR3.0 vs Context: eNpHR3.0	0.4300
	Time Effect: F (1, 13) = 0.007614		0.9318	SAD14: eYFP vs SAD14: eNpHR3.0	0.5884
	Interaction: F (1, 13) = 5.136		0.0411	Context: eYFP vs Context: eNpHR3.0	0.0001
2L (main)	Kolmogorov-Smirnov test	eYFP=353, eNpHR3.0=85			
	Kolmogorov-Smirnov D = 0.4379		<0.0001		
2L (inset)	Mann Whitney Test	eYFP=353, eNpHR3.0=85			
	Mann-Whitney U = 9635		<0.0001		
2M	Two-way ANOVA	eYFP=8, eNpHR3.0=7		SAD14: eYFP vs Context: eYFP	0.0016
	Group Effect: F (1, 13) = 16.82		0.0012	SAD14: eNpHR3.0 vs Context: eNpHR3.0	0.2869
	Time Effect: F (1, 13) = 3.392		0.0885	SAD14: eYFP vs SAD14: eNpHR3.0	0.8705
	Interaction: F (1, 13) = 21.20		0.0005	Context: eYFP vs Context: eNpHR3.0	<0.0001
2O	Two-way ANOVA	eYFP=7, eNpHR3.0=6			
	Group Effect: F (1, 11) = 9.860		0.0094		
	Time Effect: F (1.432, 15.75) = 20.36		0.0001		
	Interaction: F (1.432, 15.75) = 5.177		0.0270		
2P	Two-way ANOVA	eYFP=7, eNpHR3.0=6		Ext: eYFP vs Cue: eYFP	0.0016
	Group Effect: F (1, 11) = 6.048		0.0317	Ext: eNpHR3.0 vs Cue: eNpHR3.0	0.199
	Time Effect: F (1, 11) = 24.88		0.0004	Ext: eYFP vs Ext: eNpHR3.0	0.9728
	Interaction: F (1, 11) = 3.321		0.0957	Cue: eYFP vs Cue: eNpHR3.0	0.0238

Figure	Statisitical test	n	*p*-value	Post test	*p*-value
2Q (main)	Kolmogorov-Smirnov test	eYFP=170, eNpHR3.0=92			
	Kolmogorov-Smirnov D = 0.09118		0.7038		
2Q (inset)	Mann Whitney Test	eYFP=170, eNpHR3.0=92			
	Mann-Whitney U = 7516		0.6045		
2R	Two-way ANOVA	eYFP=7, eNpHR3.0=6		Ext: eYFP vs Cue: eYFP	0.0009
	Group Effect: F (1, 11) = 5.750		0.0354	Ext: eNpHR3.0 vs Cue: eNpHR3.0	0.2666
	Time Effect: F (1, 11) = 26.01		0.0003	Ext: eYFP vs Ext: eNpHR3.0	0.989
	Interaction: F (1, 11) = 4.880		0.0493	Cue: eYFP vs Cue: eNpHR3.0	0.0144
3E	Mixed-effects Model	n=29			
	Lever Effect: F (2, 84) = 201.8		<0.0001		
	Day Effect: F (9.565, 794.7) = 2.232		0.0161		
	Interaction: F (19.13, 794.7) = 1.135		0.3092		
3F	Correlation	n=14			
	Pearson *r* = 0.7947		0.0007		
3G (main)	Kolmogorov-Smirnov test	eYFP=353, eNpHR3.0=85			
	Kolmogorov-Smirnov D = 0.1217		0.0033		
3G (inset)	Mann Whitney Test	eYFP=353, eNpHR3.0=85			
	Mann-Whitney U = 9635		0.7091		
3H	Mixed-effects Model	n=29			
	Bout Type Effect: F (1, 56) = 392.1		<0.0001		
	Day Effect: F (8.547, 478.6) = 4.087		<0.0001		
	Interaction: F (8.547, 478.6) = 1.466		0.1619		
3I	Two-way ANOVA	eYFP=7, eNpHR3.0=5			
	Group Effect: F (1, 10) = 0.05664		0.8167		
	Time Effect: F (1.346, 13.46) = 30.75		<0.0001		
	Interaction: F (1.346, 13.46) = 0.09764		0.8308		
3J	Two-way ANOVA	eYFP=7, eNpHR3.0=5		SAD14: eYFP vs Context: eYFP	0.6612
	Group Effect: F (1, 10) = 0.2653		0.6177	SAD14: eNpHR3.0 vs Context: eNpHR3.0	>0.9999
	Time Effect: F (1, 10) = 0.7224		0.4153	SAD14: eYFP vs SAD14: eNpHR3.0	0.8531
	Interaction:F (1, 10) = 0.5989		0.4569	Context: eYFP vs Context: eNpHR3.0	>0.9999

Figure	Statisitical test	n	*p*-value	Post test	*p*-value
3K	Two-way ANOVA	eYFP=6-7, eNpHR3.0=5		SAD14: eYFP vs Context: eYFP	0.0457
	Group Effect: F (1, 10) = 0.09440		0.765	SAD14: eNpHR3.0 vs Context: eNpHR3.0	0.0139
	Time Effect: F (1, 9) = 19.47		0.0017	SAD14: eYFP vs SAD14: eNpHR3.0	0.7189
	Interaction: F (1, 9) = 0.6263		0.4491	Context: eYFP vs Context: eNpHR3.0	0.9497
3L (main)	Kolmogorov-Smirnov test	eYFP=325, eNpHR3.0=249			
	Kolmogorov-Smirnov D = 0.1167		0.0429		
3L (inset)	Mann Whitney Test	eYFP=325, eNpHR3.0=249			
	Mann-Whitney U = 35988		0.7091		
3M	Two-way ANOVA	eYFP=7, eNpHR3.0=5			
	Group Effect: F (1, 10) = 0.2539		0.6253		
	Time Effect: F (1, 10) = 3.244		0.1019		
	Interaction: F (1, 10) = 0.2539		0.6253		
3N	Two-way ANOVA	eYFP=9, eNpHR3.0=8			
	Group Effect: F (1, 15) = 0.1859		0.6725		
	Time Effect: F (1.249, 18.73) = 31.49		<0.0001		
	Interaction: F (1.249, 18.73) = 1.334		0.2725		
3O	Two-way ANOVA	eYFP=9, eNpHR3.0=8		Ext: eYFP vs Cue eYFP	0.0013
	Group Effect: F (1, 15) = 0.6103		0.3534	Ext: eNpHR3.0 vs Cue: eNpHR3.0	0.0282
	Time Effect: F (1, 15) = 29.60		<0.0001	Ext: eYFP vs Ext: eNpHR3.0	>0.9999
	Interaction: F (1, 15) = 0.8271		0.3775	Cue: eYFP vs Cue: eNpHR3.0	0.6726
3P	Two-way ANOVA	eYFP=9, eNpHR3.0=8		Ext: eYFP vs Cue eYFP	0.7994
	Group Effect: F (1, 15) = 0.01548		0.9026	Ext: eNpHR3.0 vs Cue: eNpHR3.0	0.676
	Time Effect: F (1, 15) = 2.462		0.1375	Ext: eYFP vs Ext: eNpHR3.0	0.999
	Interaction: F (1, 15) = 0.03601		0.852	Cue: eYFP vs Cue: eNpHR3.0	>0.9999
3Q (main)	Kolmogorov-Smirnov test	eYFP=238, eNpHR3.0=105			
	Kolmogorov-Smirnov D = 0.2569		0.0001		
3Q (inset)	Mann Whitney Test	eYFP=238, eNpHR3.0=105			
	Mann-Whitney U = 9196		<0.0001		
3R	Two-way ANOVA	eYFP=9, eNpHR3.0=8		Ext: eYFP vs Cue eYFP	0.0009
	Group Effect: F (1, 15) = 1.452		0.2468	Ext: eNpHR3.0 vs Cue: eNpHR3.0	0.0569
	Time Effect: F (1, 15) = 23.11		0.0002	Ext: eYFP vs Ext: eNpHR3.0	>0.9999
	Interaction:F (1, 15) = 1.667		0.2162	Cue: eYFP vs Cue: eNpHR3.0	0.1678

Figure	Statisitical test	n	*p*-value	Post test	*p*-value
S2 A	Mixed-effects Model	eYFP=8, eNpHR3.0=7		eYFP: laser on vs laser off	>0.9999
	Group Effect: F (1, 13) = 24.51		0.0003	eNpHR3.0: laser on vs laser off	0.8991
	Laser Effect: F (1, 13) = 0.3883		0.5440	Laser on: eYFP vs eNpHR3.0	0.0001
	Interaction: F (1, 13) = 0.3023		0.5971	Laser off: eYFP vs eNpHR3.0	0.0002
S2 B	One-way ANOVA	eYFP=8, med eNpHR3.0=4, lat eNpHR3.0=7		eYPF vs med eNpHR3.0	0.9706
	F (2, 16) = 9.337		0.0021	eYPF vs lat eNpHR3.0	0.0036
				med eNpHR3.0 vs lat eNpHR3.0	0.0092
S2 C	Two-way ANOVA	eYFP=7, eNpHR3.0=6		eYFP: laser on vs laser off	0.9916
	Group Effect: F (1, 11) = 4.861		0.0497	eNpHR3.0: laser on vs laser off	0.7478
	Laser Effect: F (1, 11) = 0.2940		0.5985	Laser on: eYFP vs eNpHR3.0	0.0902
	Interaction: F (1, 11) = 1.176		0.3014	Laser off: eYFP vs eNpHR3.0	0.3339
S3 A	Two-way ANOVA	eYFP=6, eNpHR3.0=5		eYFP: laser on vs laser off	0.3835
	Group Effect: F (1, 10) = 0.009376		0.9248	eNpHR3.0: laser on vs laser off	0.3419
	Laser Effect: F (1, 10) = 0.07256		0.7931	Laser on: eYFP vs eNpHR3.0	0.959
	Interaction: F (1, 10) = 6.278		0.0311	Laser off: eYFP vs eNpHR3.0	0.9895
S3 B	Two-way ANOVA	eYFP=9, eNpHR3.0=8		eYFP: laser on vs laser off	0.9374
	Group Effect: F (1, 15) = 0.7371		0.4041	eNpHR3.0: laser on vs laser off	0.9975
	Laser Effect: F (1, 15) = 0.4691		0.5038	Laser on: eYFP vs eNpHR3.0	0.9332
	Interaction: F (1, 15) = 0.06952		0.7956	Laser off: eYFP vs eNpHR3.0	0.8504
