# Supplementary material for: Activation of Insula‐Accumbal Projection Neurons Is Required for Relapse‐Like Behaviour Following Opioid Self‐Administration
Source: Addict Biol. 2026 Jan 12;31(1):e70118. doi: 10.1111/adb.70118 (PMC12795779; doi:10.1111/adb.70118)
Supplement: Supplementary file 2 — Figure S1: adb_70118‐sup‐0002‐Supplement.docx. Injection site mapping for Ai‐14 tracing study. (A) Placements for lateral NAcc injection sites (n = 3). (B) Placements for medial NAcc injection sites (n = 2). NAcc, nucleus accumbens core. Figure S2: Supplemental data for heroin self‐administration experiment. (A) Active presses during both laser on and laser off epochs were significantly higher in the eYFP group compared to the eNpHR3.0 group during the context test (n = 7–8 mice per group; mixed‐effects model, group effect: F (1, 13) = 24.51, p = 0.0003, laser effect: F (1, 13) = 0.3883, p = 0.5440, interaction: F (1, 13) = 0.3023, p = 0.5917, Sidak's post hoc test, laser on: p = 0.0001, laser off: p = 0.0002). (B) Total active lever presses during context test did not differ compared to eYFP when optical fibres were targeted to the medial NAcc and were significantly higher compared to when the aIC➔lateral NAcc pathway was targeted (Data for eYFP and lateral NAcc eNpHR3.0 also appear in Figure 2K; n = 4–8, one‐way ANOVA, F (2, 16)=9.337, p = 0.0021, Tukey's post hoc test, eYFP versus Med eNpHR3.0: p = 0.9706 eYFP versus Lat eNpHR3.0: p = 0.0036, Med eNpHR3.0 versus Lat eNpHR3.0: p = 0.0092). (C) Active presses during laser on and laser off epochs were higher in the eYFP group compared to the eNpHR3.0 group during cue reinstatement (n = 6–7 per group, two‐way ANOVA, group effect: F (1, 11) = 4.861, p = 0.0497, laser effect: F (1, 11) = 0.2940, p = 0.5985, interaction: F (1, 11) = 1.176, p = 0.3014). (D) Viral expression of eYFP and eNpHR3.0‐eYFP (top) and lateral (purple) or medial (red) optical fibre placement (bottom) for animals in heroin self‐administration paradigm (n = 17 lateral NAcc, n = 4 medial NAcc). Data are mean ± SEM **p < 0.01; ***p < 0.001. Lat, lateral; Med, medial; NAcc, nucleus accumbens core. Figure S3: Supplemental data for sucrose self‐administration experiment. (A) There was a small but significant interaction between laser status and experimen [file ADB-31-e70118-s002.docx]

**
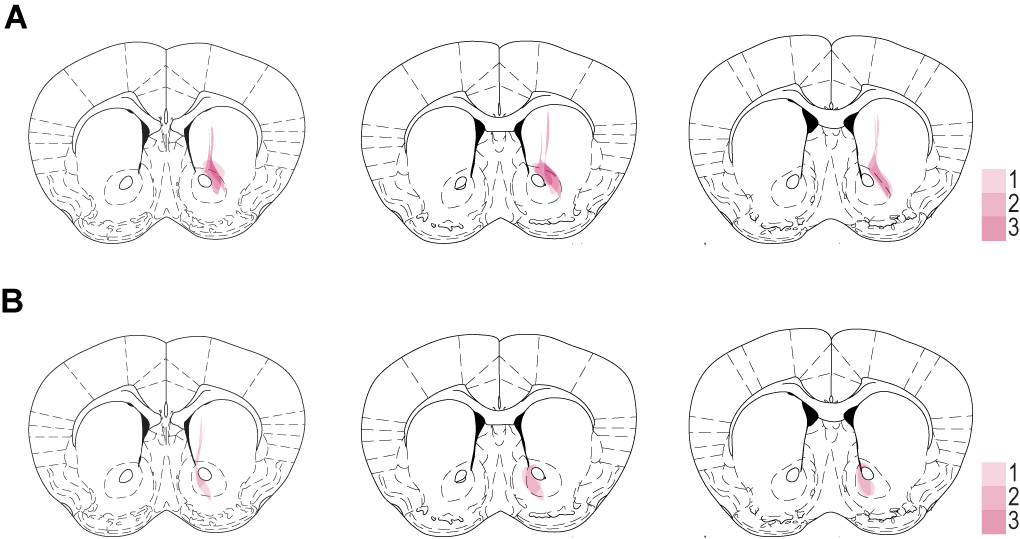
Figure S1**

**Figure S1. Injection site mapping for Ai-14 tracing study.** (**A**) Placements for lateral NAcc injection sites (n=3). (**B**) Placements for medial NAcc injection sites (n=2). NAcc, Nucleus Accumbens Core.

**
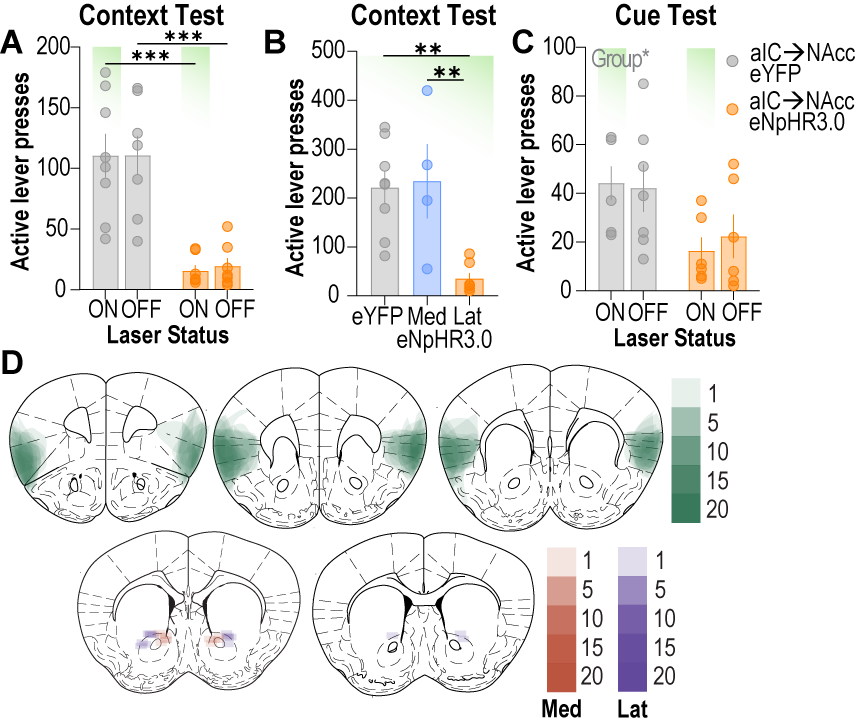
Figure S2**

**S2. Supplemental data for heroin self-administration experiment.** (**A**) Active presses during both laser on and laser off epochs were significantly higher in the eYFP group compared to the eNpHR3.0 group during the context test (n=7-8 mice per group; mixed-effects model, group effect: F (1, 13)=24.51, p=0.0003, laser effect: F (1, 13)=0.3883, p=0.5440, interaction: F (1, 13) = 0.3023, p=0.5917, Sidak’s post-hoc test, laser on: p=0.0001, laser off: p=0.0002). (**B**) Total active lever presses during context test did not differ compared to eYFP when optical fibers were targeted to the medial NAcc, and were significantly higher compared to when the the aIC🡪lateral NAcc pathway was targeted (Data for eYFP and lateral NAcc eNpHR3.0 also appear in figure 2K ; n=4-8, one-way ANOVA, F_(2,16)_=9.337, *p*=0.0021, Tukey’s post hoc test, eYFP vs Med eNpHR3.0: *p*=0.9706 eYFP vs Lat eNpHR3.0: *p*=0.0036, Med eNpHR3.0 vs Lat eNpHR3.0: *p*=0.0092). (**C**) Active presses during laser on and laser off epochs were higher in the eYFP group compared to the eNpHR3.0 group during cue reinstatement (n=6-7 per group, two-way ANOVA, group effect: F (1, 11)=4.861, p=0.0497, laser effect: F (1, 11)=0.2940, p=0.5985, interaction: F (1, 11)=1.176, p=0.3014). (**D**) Viral expression of eYFP and eNpHR3.0-eYFP (top) and lateral (purple) or medial (red) optical fiber placement (bottom) for animals in heroin self-administration paradigm (n=17 lateral NAcc, n=4 medial NAcc). Data are mean ± SEM. ***p*<0.01; ***p<0.001. Lat, lateral; Med, medial; NAcc, Nucleus Accumbens Core.

**Figure S3**


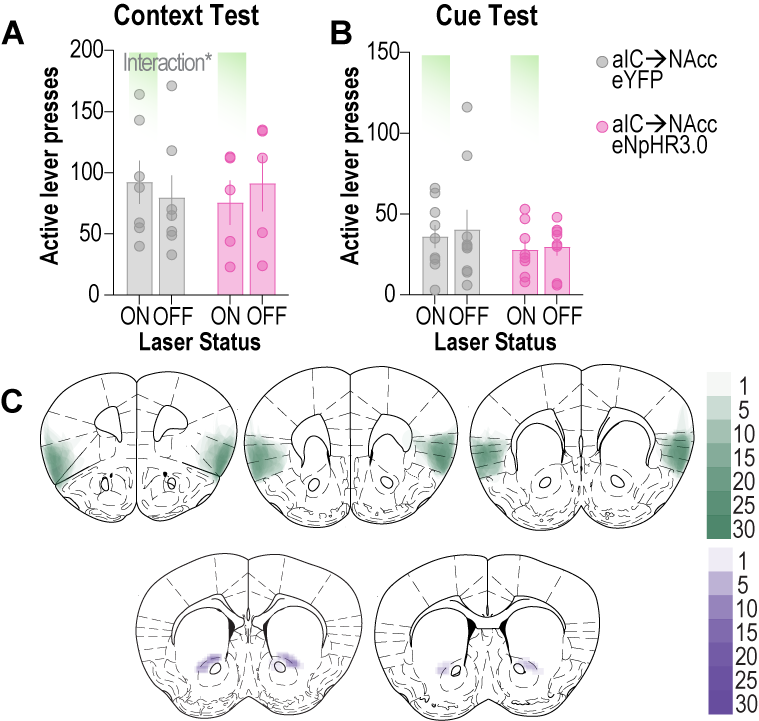


**S3. Supplemental data for sucrose self-administration experiment.** **A**) There was a small but significant interaction between laser status and experimental group in active lever presses during the context test (n=5-7, mixed-effects analysis, group: F (1, 10)=0.009376, p=0.9248, laser status: F (1, 10) = 0.07256, p=0.7931, interaction: F (1, 10) = 6.278, p=0.0311). (**B**) Active presses during laser on and laser off epochs did not differ between eYFP and eNpHR3.0 groups during cue reinstatement. (**C**) Viral expression of eYFP and eNpHR3.0-eYFP (top) and optical fiber placement (bottom) for animals in sucrose self-administration paradigm (n=29). NAcc, Nucleus Accumbens Core.
